# Optimal Tranexamic Acid Dosing for Adolescent Idiopathic Scoliosis Surgery: A Frequentist Network Meta-Analysis

**DOI:** 10.1097/BRS.0000000000005465

**Published:** 2025-08-04

**Authors:** Paweł Łajczak, Ayesha Ayesha, Aisha Rizwan Ahmed, Paweł Chochoł, Enzo von Quednow, Fabio Victor Vieira Rocha, Oguz Kagan Sahin, Numa Rajab, Martin Kotochinsky, Yasmin Picanço Silva, Yan Gabriel Morais David Silva, Walter Fagundes

**Affiliations:** a Faculty of Medical Sciences in Zabrze, Medical University of Silesia, Katowice, Poland; b Shifa College of Medicine, Islamabad, Pakistan; c Jinnah Medical and Dental College, Karachi, Sindh, Pakistan; d Department of Neurology and Neurosurgery, University of Warmia and Mazury, Olsztyn, Poland; e Department of Clinical Neurophysiology, Hospital Universitari Sant Joan de Reus, Reus, Spain; f Division of Neurosurgery, Federal University of Espírito Santo (UFES), Vitória, Espirito Santo, Brazil; g Edremit State Hospital, Balikesir, Turkey; h Sulaiman AlRajhi University, Al-bukairiyah, Qassim, Saudi Arabia; i National University of Cuyo, Mendoza, Argentina; j Healthcare Institution of South Iceland, Selfoss, Iceland; k Spine Surgery, Hospital Ortopédico do Estado (Soc. Ben. Israelita Albert Einstein), Salvador, Bahia, Brazil

**Keywords:** adolescent idiopathic scoliosis, blood loss, network meta-analysis, spinal fusion, tranexamic acid

## Abstract

**Study Design.:**

Systematic review and network meta-analysis (NMA).

**Objective.:**

This NMA evaluates the clinical efficacy and safety of various doses of Tranexamic acid (TXA) for adolescent idiopathic scoliosis (AIS) instrumentation.

**Summary of Background Data.:**

TXA is an antifibrinolytic agent widely used in spine surgery. However, the optimal TXA dose remains unclear, especially for younger patients.

**Methods.:**

We performed a systematic search across PubMed, Scopus, Web of Science, and the Cochrane Library up to March 2025. We included studies that compared different TXA doses or patients without TXA in AIS surgery. We evaluated five TXA doses: TXA 0 (control without TXA); TXA 1 (10 mg/kg bolus + 1 mg/kg/hour infusion); TXA 2 (15–20 mg/kg bolus + 1–10 mg/kg/hour infusion); TXA 3 (30–50 mg/kg bolus of TXA + 5–10 mg/kg/hour infusion); and TXA 4 (100 mg/kg bolus of TXA + 10 mg/kg/hour infusion). This meta-analysis compared bleeding volume, surgical time, transfusion rates, length of hospital stay, and complications. Random-effects frequentist NMA was employed. Treatments were ranked with P-scores.

**Results.:**

A total of 1523 patients, mainly aged 13 to 18 years from 16 studies were included in this analysis (eight retrospective studies). TXA 4 outperformed other doses in reducing intraoperative bleeding, operation time, and cell saver volume. TXA 3 also showed significant benefits but was less effective than TXA 4. There was no difference in complication rates, although these data were not uniformly reported in the studies. No evidence of publication bias was found. Quality concerns were observed.

**Conclusion.:**

The tranexamic acid dose of 100 mg/kg bolus + 10 mg/kg/hour infusion (TXA 4) was the most effective in bleeding control during AIS instrumentation in teenagers. While these results are promising, more randomized trials are needed to directly compare various TXA doses.

Adolescent idiopathic scoliosis (AIS) remains the most common type of pediatric scoliosis. The prevalence of AIS is around 1% to 3% in the population, with most cases best treated using conservative measures (physiotherapy and bracing are the most frequently used). Only a few patients undergo surgery. The standard surgical treatment involves Posterior Spinal Fusion (PSF) with pedicle screws, typically for spinal curvature greater than 45 to 50°.^1^

However, due to the need for high accuracy during instrumentation, various complications may arise associated with the instrumentation of the AIS spine, along with vascular complications and bleeding, which remain significant concerns during the procedure.^2^ Literature reports that excessive perioperative bleeding may be linked to factors such as female sex, low preoperative hemoglobin, lumbar section involvement, increased Cobb angle, or multiple spinal segment fusions.^3^ Excessive bleeding during the perioperative period can lead to additional complications for patients, increased morbidity, the need for further transfusions during procedures, or even increased mortality.^4^ Additional transfusions may lead to a higher risk of alloimmunization reactions, which are significantly more common in younger patient populations, potentially resulting in increased costs within the medical system.

Intraoperative bleeding has long been a challenge in the surgical treatment of AIS. Over the past years, surgeons and anesthesiologists have explored various methods to reduce blood loss and improve outcomes. Techniques involved maintaining normothermia, applying regional anesthesia during the procedure, inducing hypotension, and more. With time, advances in surgical techniques, including minimally invasive surgery and the application of robotic and computer navigation workstations, have provided new benefits for controlling blood loss in managing AIS.^5^ Pharmacological solutions have been widely studied alongside these methods, including antifibrinolytic agents such as apoprotein, aminocaproic acid, and tranexamic acid (TXA).^6^ TXA utilizes its antifibrinolytic effects to inhibit the interaction of plasminogen with formed plasmin and fibrin, thereby stabilizing the fibrin meshwork formed during secondary hemostasis.^7^ This mechanism explains its well-known association with reducing intraoperative and perioperative bleeding during procedures, consequently lowering the need for transfusions.^8,9^

It has been extensively studied in surgical fields, including spine surgery, particularly for managing various degenerative disorders of the spine, such as laminectomy or spinal fusion procedures of vertebrae.^10^ While TXA remains effective in AIS procedures, the optimal dosing remains controversial.^11^ Currently, there is a lack of network meta-analyses comparing different doses of TXA; therefore, this study aims to evaluate these doses to find the most clinically effective regimen. We aim to focus on blood loss and other hematological perioperative outcomes.

## MATERIALS AND METHODS

This systematic review followed the Cochrane Handbook for conducting systematic reviews and meta-analyses.^12^ The reporting of this study complied with the Preferred Reporting Items for Systematic Literature Reviews and Meta-Analyses (PRISMA) guidelines, including an extension for network meta-analyses (NMA).^13^ A prospective protocol registration was conducted in the Prospective Register of Systematic Reviews (PROSPERO) database - CRD420251013894. This study did not require ethical approval due to its review nature.

### Search Strategy

We systematically searched the PubMed, Scopus, Web of Science, and Cochrane Library databases from inception to March 2025. Search terms included medical subject headings [MeSH]/EMTREE terms and text words: “scoliosis,” “adolescent idiopathic scoliosis,” “AIS,” “idiopathic scoliosis,” “tranexamic acid,” “TXA,” “transamine,” and “cyklokapron.” The references of all relevant studies were manually searched to identify additional eligible studies.

### Study Selection Criteria

Four authors independently screened titles and abstracts and thoroughly evaluated the studies for eligibility. Discrepancies were resolved through discussions with a senior author. Inclusion was limited to studies that met all the following criteria: (1) randomized controlled trials or observational studies; (2) comparing the usage of TXA at different doses or to placebo/control without TXA; (3) enrolling or reporting data on patients undergoing spine surgery for AIS; and (4) reporting at least one outcome of interest. Outcomes in the NMA included intraoperative blood loss (blood loss during the procedure only), postoperative drain blood loss, total blood loss (the sum of intraoperative and postoperative blood loss), intraoperative blood loss per vertebral level, operation time, length of hospitalization, intraoperative cell saver transfusion volume, intraoperative and postoperative allogenic transfusions, and complications.

The exclusion criteria were: (1) no control group (single-arm studies); (2) patients undergoing spine surgery for conditions other than AIS (eg, different types of pediatric scoliosis, including congenital or neuromuscular); (3) duplicated publications of previously reported data to prevent overlapping populations; (4) non-English publications; and (5) case reports, case series, or conference abstracts.

### Screening and Data Extraction

Three authors independently extracted data using Microsoft Excel software (Microsoft Corp., Redmond, WA, United States), adhering to predefined search criteria and quality assessment guidelines. Any disagreements were resolved through consensus with the senior authors. The extracted data included the authors and publication year, the years of the study period, country, study design (observational or RCT), population characteristics (number of patients, sex, age, weight) for each study arm, and TXA dose(s).

### Treatment Arm Classification and Outcomes

This NMA compared five different dosage groups:

TXA 0 (Placebo)—In this group, patients did not receive any TXA for the AIS surgery.
TXA 1—Patients were administered a 10 mg/kg bolus of TXA and a 1 mg/kg/hour infusion.
TXA 2—Patients were administered a 15 to 20 mg/kg bolus of TXA and a 1 to 10 mg/kg/hour infusion.
TXA 3—Patients were administered a 30 to 50 mg/kg bolus of TXA and a 5 to 10 mg/kg/hour infusion. This group was later split into TXA 3A (30 mg/kg bolus) and TXA 3B (50 mg/kg bolus).
TXA 4—Patients were administered a 100 mg/kg bolus of TXA and a 10 mg/kg/hour infusion.

### Frequentist Network Meta-Analysis

The netmeta, pcnetmeta, meta, ggplot, and metafor packages were employed to run frequentist NMA in R studies.^14–18^ REML estimator was applied for all analyses, with a random-effects model. Ninety-five CIs (95% CI) were employed and presented with odds ratios (OR) or mean differences (MD) in the brackets. Heterogeneity was assessed with I^2^, and a value exceeding 50% was considered significant. Network plots and league tables were employed to visualize NMA results. The league table displays comparisons between interventions listed in rows and columns. Each cell shows the effect size, 95% CI, and *P*-value for the row intervention compared with the column intervention.

Ranking of treatment arms was performed with p-scores—a p-score of 1 is considered the best treatment for given outcome. Median conversion was employed with methods described by Wan and Lou.^19,20^

#### Sensitivity Analysis

Sensitivity analysis was performed by splitting group TXA 3 into TXA 3A (30 mg/kg bolus) and TXA 3B (50 mg/kg bolus) as described above.

In addition, due to inclusion of studies with PSF-only approach and with mixed surgical approaches (including hybrid anterior/posterior), a sensitivity analysis on PSF-only approach was performed. Due to the fact, that majority of outcomes were reported in PSF-only studies, this was only performed for intraoperative blood loss and complications, where studies with mixed approaches were present.

### Quality Assessment

Nonrandomized studies were assessed using the ROBINS-I tool, while RCT trials were evaluated using the RoB2 Cochrane tool. In both tools, two reviewers independently performed quality assessment, and three other authors resolved potential conflicts. Authors assessed seven domains in ROBINS-I and five domains in RoB2, which are described in greater detail in the respective references.^21,22^ Visualization of quality assessment results was performed with robvis R tool.^23^ Finally, Egger test of asymmetry was employed to investigate potential publication bias in the outcomes, with at least 10 studies, as suggested by Cochrane Collaboration.^24^

## RESULTS

**Figure 1:**
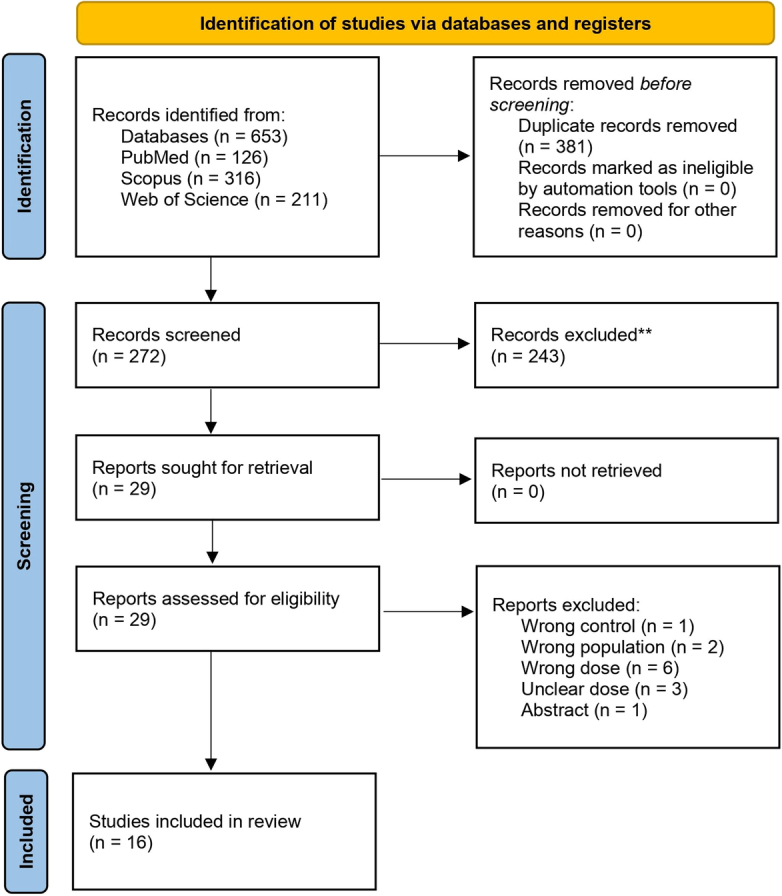
PRISMA flow diagram.

A systematic search was conducted from inception to March 2025. Following this search, 16 studies were included in the meta-analysis.^10,25–39^ Detailed results of the exclusion and full screening process stages are shown in Figure 1.

## Baseline Characteristics of Included Studies

This meta-analysis comprised 16 studies, including 6 randomized trials. Most studies were conducted in the USA (n = 7). A total of 1523 patients were included in this synthesis. Thirteen studies reported a TXA 0 group (661 patients), 7 studies reported a TXA 1 group (307 patients), 3 studies reported a TXA 2 group (122 patients), 6 studies reported a TXA 3 group (295 patients), and 3 studies reported a TXA 4 group (138 patients). The PSF approach was present in all studies, while 2 studies additionally provided PSF and anterior spinal fusion approaches. Detailed baseline characteristics are available in Table 1.

**TABLE 1:** Baseline Characteristics of Included Studies

Author (year)	Study design	Place, duration of study	Surgical method	No. participants	TXA Dose(s)	Age (years)†	Female (%)	Weight (kg)†	Number of levels fused†	Duration of surgery (minutes)†	Preoperative Hb (g/dL)†
Berney (2015)^25^	Retrospective chart review	Ireland, 2009–2013	PSF	TXA (2) 31/TXA (0) 25	15 mg/kg + 10 mg/kg/hour (TXA 2)	15.3/16.4	68.8/60	N/A	12.7/12.7	188/223	13.5/13.2
Bosch (2019)^26^	Prospective cohort	United States, 2012–2014 and 2016–2017	PSF	TXA (3) 30/TXA (0) 58	30 mg/kg + 10 mg/kg/hour (TXA 3)	13.7±1.6/13.5±1.7	86.7/81	N/A	11.1±1.6/11.1±1.7	220±52/203±47	N/A
Ezhevskaya (2018)^27^	Prospective double-blind cohort design	Russia, 2015–2017	PSF	TXA (2) 80/TXA (0) 80	15 mg/kg + 1 mg/kg/hour (TXA 2)	15.4±1.2/16±0.7	N/A/95	N/A	12.8±5.5/12.8±6.1	218.1±12.7/224.5±11.2	13.1±1.0/12.9±0.8
Goobie (2018)^10^	RCT	United States	PSF	TXA (3) 56/TXA (0) 55	50 mg/kg + 10 mg/kg/hour (TXA 3)	14.9±2.0/14.7±1.8	82.1/83.6	55.1±11.8/57.6±11.9	10 (5–13)/9 (5–13)	264 ± 81/266 ± 65	13.6±1.0/13.5±1.1
Grant (2009)^28^	Retrospective chart review	Canada, 2005–2006	PSF	TXA (1) 15/TXA (2) 11	10 mg/kg + 1 mg/kg/hour (TXA 1)/20 mg/kg + 10 mg/kg/h (TXA 2)	14.7±2.0/15.4±2.3	87/100	50.3±8.6/58.0±10.8	9±2/9±2	6.4±1.6* (hours)/7.2±1.1*	134.1±12.1¶/138.2±17.3¶
Johnson (2017)^29^	Retrospective medical records review	United States, 2009–2015	PSF	TXA (1) 71/TXA (3) 44	10 mg/kg + 1 mg/kg/hour (TXA 1)/50 mg/kg + 5 mg/kg/h (TXA 3)	14.3±1.9/14.5±1.9	69/65.9	52±16/54±16	10.5± 2.9/10.9± 2	3.6±0.9*/3.6 ±0.7*	13.7±1.3/13.5±1.5
Jones (2017)^30^	Retrospective cohort	United States, 2011–2014	PSF	TXA (1) 18/TXA (0) 18	10 mg/kg + 1 mg/kg/hour (TXA 1)	16.1± 3.1/15.2± 3.4	88.9/83.3	N/A	10.5± 2.2/10.5± 2.2	6.1± 1.1*/6.1± 1.2*	13.9± 1.3/13.9± 1.1
Ng (2015)^31^	Retrospective	China, 2005–2010	PSF	TXA (4) 55/TXA (0) 35	100 mg/kg + 10 mg/kg/hour (TXA 4)	15.16±2.61/15.31±2.97	100/100	45.25±8.96/42.88±7.76	13.51±1.62/12.14±2.79	436.71±122.53/502.14±85.81	NA
Ngo (2013)^32^	Retrospective	United States, 2003–2010	PSF	TXA (3) 70/TXA (0) 160	50 mg/kg + 5 or 10 mg/kg/hour (TXA 3)	14.8±2.6/14.8±2	76/78	56.5 (49–68)/55.1 (46–63)∥	10 (8–12)/10 (9–11)∥	237 (198–270)/230 (203–269)∥	NA
Ramkiran (2020)^33^	RCT	India	ASF or PSF	TXA (3) 12/TXA (0) 12	50 mg/kg + 10 mg/kg/hour (TXA 3)	12.83±3.689/13.5±1.883	66.7/91.7	30.25±10.306/37.67±5.742	NA	NA	13.14±1.18/13.18±1.52
Saleh (2018)^34^	RCT	Egipt, 2017	Combined ASF+ PSF or PSF	TXA (1) 25/TXA (0) 25	10 mg/kg + 1 mg/kg/hour (TXA 1)	14.6±2.1/14.6±2.16	44/44	39±3.97/39.4±4.3	NA	188±13.41/208.3±21.1	NA
Sethna (2004)	RCT	United States	PSF	TXA (4) 12/TXA (0) 21	100 mg/kg + 10 mg/kg/h (TXA 4)	13.6±1.8 / 14.0±2.0	26.1/38.1	59.4±18.3/52.4±15.7	14 (9–16)/13 (7–18)§	396±108/366±108	NA
Sui (2016)^36^	Retrospective	China, 2011–2015	PSF	TXA (4) 71/TXA (0) 66	100 mg/kg + 10 mg/kg/hour (TXA 4)	15.5 / 16.2	69/68.2	NA	13.1/12.8	209 / 215	13.2/13.4
Verma (2014)^37^	RCT	United States	PSF	TXA (1) 36/TXA (0) 47	10 mg/kg+1 mg/kg/hour (TXA 1)	15.5±2.37/15.01±2.37	88.9/65.96	NA	8.8±2.3/9.0±2.0	NA	NA
Hasan (2021)^38^	RCT	Malaysia, 2017–2018	PSF	TXA (3) 83/TXA (1) 83	30 mg/kg +10 mg/kg/hour (TXA 3)/10 mg/kg + 1 mg/kg/hour (TXA 1)	14.1±2.1/14.6±3.0	91.6/81.9	45.3±9.0/46.6±9.3	11 (10–13)/111 (10–12)§	130 (105–155)/120 (100–150)§	13.8±1.0/13.8±1.1
Lebedeva (2016)^39^	Retrospective	Russia Federation, 2012–2015	PSF	TXA (1) 58/TXA (0) 70	10 mg/kg+1 mg/kg/hour (TXA 1)	18.6±7.0/18.3±5.7	NA	53.1±9.5/51.0±10.5	3.8±1.0/4.0±1.2	169.0±30.0/168.0±30.0	NA

AIS indicates Adolescent Idiopathic Scoliosis; PSF, posterior spinal fusion; ASF, anterior spinal fusion; TXA, tranexamic acid; C, control.

* Hours,

† Mean±SD except ^a,b,c^.

§ Median (range).

∥ Median (IQR).

¶ g/L.

### Network Meta-Analysis Results

#### Intraoperative Blood Loss

**Figure 2:**
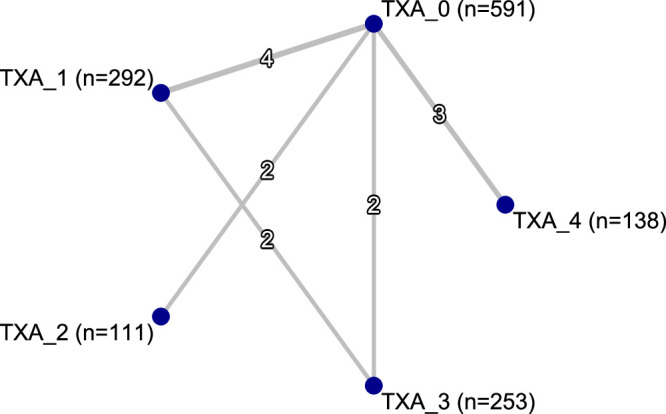
Network plot for intraoperative blood loss.

**Figure 3:**
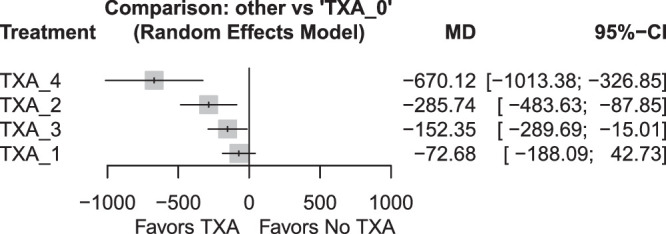
Forest plot for intraoperative blood loss.

**Figure 4:**
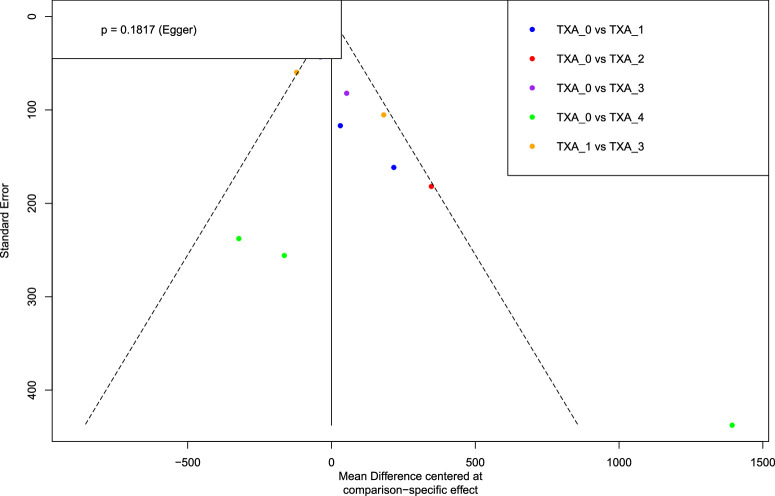
Funnel plot for intraoperative blood loss.

Regarding intraoperative blood loss, all TXA doses except TXA 1 [TXA 0 vs. TXA 1 - MD 72.68 (95% CI −42.73; 188.09); *P* = 0.2171] demonstrated a statistically significant reduction in blood loss compared with the TXA 0 group (Table 2; Figure 2 and Figure 3; *P* < 0.05). The TXA 1 [MD 597.44 (95% CI 235.29; 959.59); *P* = 0.0012] and TXA 3 [517.77 (148.04; 887.49); *P* = 0.0061] groups were also inferior to the TXA 4 arm. The heterogeneity was significant (*P* = 0.0007), and I^2^ of the outcome was 68.7%. The Egger test showed no substantial evidence of publication bias (Figure 4; *P* = 0.182).

**TABLE 2:** League Table for Intraoperative Blood Loss

TXA 0				
72.68 (−42.73; 188.09); *P* = 0.2171	TXA 1			
285.74 (87.85; 483.63); *P* = 0.0047	213.07 (−16.02; 442.15); *P* = 0.0683	TXA 2		
152.35 (15.01; 289.69); *P* = 0.0297	79.67 (−61.42; 220.77); *P* = 0.2684	−133.39 (−374.27; 107.49); *P* = 0.2778	TXA 3	
670.12 (326.85; 1013.38); *P* = 0.0001	597.44 (235.29; 959.59); *P* = 0.0012	384.37 (−11.85; 780.60); *P* = 0.0573	517.77 (148.04; 887.49); *P* = 0.0061	TXA 4

Results are presented as mean differences.

Sensitivity analysis on PSF-only studies yielded similar conclusions; however, TXA 2 group became significantly inferior to TXA 4 [Table 1 Supplementary Digital Content 1 (SDC), http://links.lww.com/BRS/C791].

#### Postoperative Blood Loss

TXA groups showed a significant reduction of postoperative blood loss compared with TXA 0 group (Table 3; *P* < 0.0001). TXA 2 was also inferior to the TXA 1 group, and these two groups were both significantly inferior to TXA 3 cohort. Low heterogeneity was observed (I^2^ = 0%).

**TABLE 3:** League Table for Postoperative Blood Loss

TXA 0			
147.68 (102.62; 192.74); *P* < 0.0001	TXA 1		
33.15 (20.88; 45.42); *P* < 0.0001	−114.53 (−161.23; −67.83); < 0.0001	TXA 2	
166.24 (119.21; 213.26); *P* < 0.0001	18.56 (1.47; 35.65); *P* = 0.0333	133.09 (84.49; 181.69); *P* < 0.0001	TXA 3

Results are presented as mean differences with 95% CI

#### Total Blood Loss

Regarding overall blood loss, TXA 1 and TXA 3 groups showed significant blood loss reduction compared with TXA 0 group (Table 2 SDC, Supplemental Digital Content 2, http://links.lww.com/BRS/C792). However, other doses did not report this outcome (including TXA 4 and TXA 2). There was no significant difference between TXA 1 and TXA 3 groups. Low heterogeneity was observed (I^2^ = 20.5%).

#### Intraoperative Blood Loss per Level

Regarding intraoperative blood loss per vertebral level, TXA 4 group significantly lower blood loss compared to other treatment groups (Table 3 SDC, Supplemental Digital Content 3, http://links.lww.com/BRS/C793). Other comparisons were insignificant. High heterogeneity was observed, I^2^ = 80.4%.

#### Operative Time

The TXA 4 group exhibited a shorter operation time compared with other groups (Table 4). There was no significant difference in operation times between other TXA groups. Moderate heterogeneity was observed in the analysis, with I² = 51.3%. No publication bias was detected in the study (*P* = 0.189).

**TABLE 4:** League Table for Operation Time

TXA 0				
9.94 (−0.06; 19.95); *P* = 0.0514	TXA 1			
9.02 (−5.14; 23.18); *P* = 0.2118	−0.92 (−17.98; 16.13); *P* = 0.9156	TXA 2		
−0.46 (−11.78; 10.86); *P* = 0.9366	−10.40 (−21.17; 0.37); *P* = 0.0583	−9.48 (−27.46; 8.50); *P* = 0.3013	TXA 3	
65.43 (19.63; 111.23); *P* = 0.0051	55.49 (8.60; 102.37); *P* = 0.0204	56.41 (8.47; 104.35); *P* = 0.0211	65.89 (18.71; 113.07); *P* = 0.0062	TXA 4

Results are presented as mean differences with 95% CI.

#### Length of Hospitalization

The TXA 1 arm had a longer hospitalization duration than the TXA 2 group (Table 4 SDC, Supplemental Digital Content 4, http://links.lww.com/BRS/C794). Other results were insignificant. Low heterogeneity was observed (I^2^ = 0%).

#### Volume (mL) of Intraoperative Cell Saver Transfusion

The TXA 0 group had a greater need for intraoperative cell saver transfusion than any TXA group (Table 5 SDC, Supplemental Digital Content 5, http://links.lww.com/BRS/C795). Interestingly, the TXA 2 group recorded a lower cell saver volume than the TXA 3 group. Both the TXA 2 and TXA 3 groups had higher cell saver volumes compared with the TXA 4 group. Low heterogeneity was observed (I^2^ = 0%).

#### Intraoperative Allogenic Transfusion Rate

The TXA 0 and TXA 1 groups had higher intraoperative allogenic transfusion rates than the TXA 3 group (Table 6 SDC, Supplemental Digital Content 6, http://links.lww.com/BRS/C796). Low heterogeneity was observed (I^2^ = 1.4%).

#### Postoperative Allogenic Transfusion Rate

The TXA 0 group exhibited a significantly higher postoperative allogenic transfusion rate than the TXA 3 group (Table 7 SDC, Supplemental Digital Content 7, http://links.lww.com/BRS/C797). Low heterogeneity was observed in the analysis (I^2^ = 0%).

#### Complications

There were no significant differences in complications (Table 8 SDC, Supplemental Digital Content 8, http://links.lww.com/BRS/C798). A total of 8 complications occurred, including 3 in the TXA 0 arm (2 related to hemostasis and 1 excessive bleeding), 3 in the TXA 3 arm (2 infections and 1 respiratory morbidity), and 2 in the TXA 1 group (2 respiratory morbidities). Low heterogeneity was observed, I^2^ = 0%.

Similar conclusions were seen in PSF-only sensitivity analysis with no significant differences (Table 9 SDC, Supplemental Digital Content 9, http://links.lww.com/BRS/C799).

#### Network Meta-Analysis Summary

A summary of the network meta-analysis is available in Table 5. The TXA 4 group achieved the highest P-scores in intraoperative blood loss, blood loss per level, operation time, and cell saver transfusion. The TXA 3 group achieved the highest P-scores for total blood loss, postoperative blood loss, and allogeneic transfusion rates (both intraoperative and postoperative). The TXA 2 group recorded the highest P-scores in length of hospitalization and complications. In evidence splitting, there was no significant difference between direct and indirect evidence in all analyses. In addition, no evidence of publication bias was found in the analyses.

**TABLE 5:** Summary of P-scores Across all Outcomes

Outcome	Heterogeneity, %	TXA 0	TXA 1	TXA 2	TXA 3	TXA 4
Intraoperative blood loss	68.70	0.0314	0.2651	0.7133	0.4982	**0.9919**
Total blood loss	20.50	0	0.7022	N/A	**0.7978**	N/A
Postoperative blood loss	0.00	0	0.6722	0.3333	**0.9944**	N/A
Blood loss per level	80.40	0.0422	0.4377	N/A	0.5202	**1**
Operation time	51.30	0.1665	0.6244	0.5529	0.1628	**0.9934**
Length of hospitalization	0	0.4072	0.0927	**0.9583**	0.5419	N/A
Volume (mL) of intraoperative cell saver transfusion	0	0.0003	N/A	0.6667	0.333	**1**
Intraoperative allogenic transfusion rate	1.40	0.4034	0.0713	0.6956	**0.8297**	N/A
Postoperative allogenic transfusion rate	0	0.3272	0.1885	N/A	**0.9842**	N/A
Complications	0	0.3688	0.6118	**0.7466**	0.2909	0.4819

Bold values show the highest P-score for given outcome.

Results are presented as mean differences with 95% CI.

#### Six-Arm Sensitivity Analysis

Group TXA 3 was split and analyzed into two subgroups, TXA 3A and TXA 3B (described in the methods), in a 6-arm analysis. The TXA 3A group (lower dose) showed higher P-scores in complications and postoperative blood loss compared with 3B. However, TXA 3B showed superiority in operation time, intraoperative and total blood loss, and blood loss per level compared with the 3A group. A summary of the sensitivity analysis is available as Table 10 SDC, Supplemental Digital Content 10, http://links.lww.com/BRS/C800.

#### Quality Assessment

In the ROB2 tool, three randomized trials were assessed with moderate concerns, while the remaining RCT studies were graded as having a low risk of bias. Concerns primarily arose in outcome measurement.

**Figure 5:**
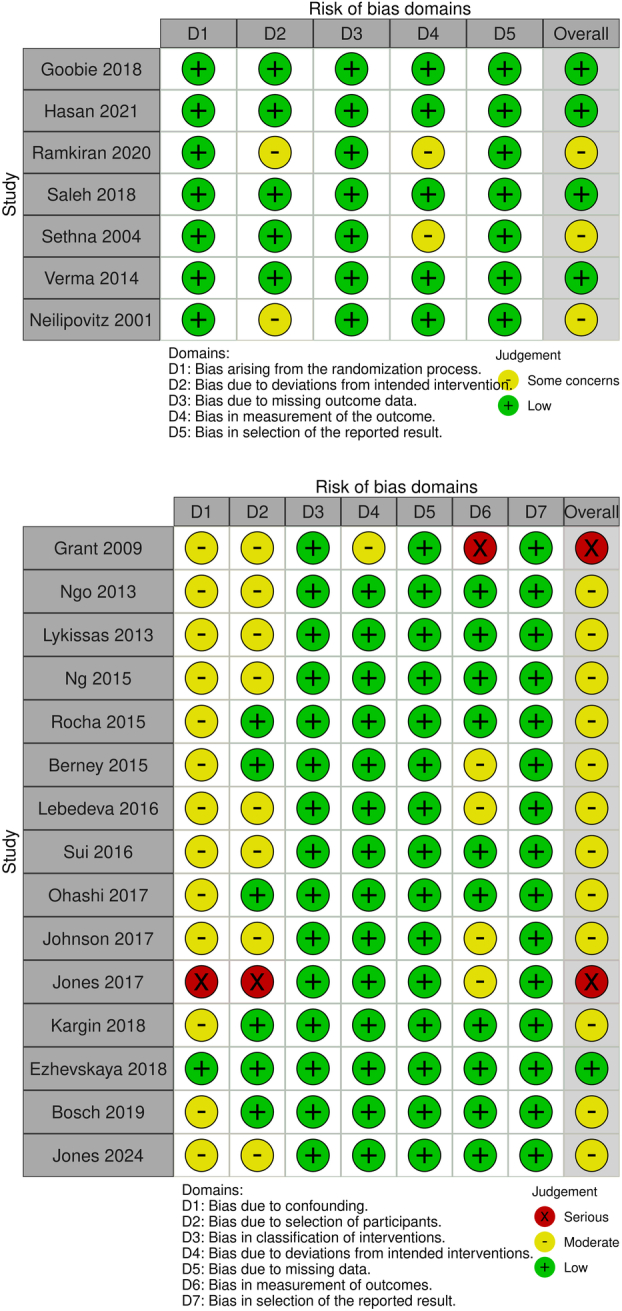
Quality assessment summary—ROB2 (upper part) and ROBINS-I (bottom part).

In the ROBINS-I tool, only one study was graded as having a low risk of bias. The other studies were assigned moderate concerns, and two were marked with a serious risk of bias. Most risk concerns were found in patient selection, group confounding, and outcome measurement. A summary of the quality assessment is available in Figure 5.

## DISCUSSION

This NMA evaluated the clinical efficacy and safety of various doses of TXA for adolescent idiopathic scoliosis spinal instrumentation in terms of bleeding control. The TXA 4 group (100 mg/kg bolus + 10 mg/kg/hour infusion) is the most effective dose. It demonstrated the highest efficacy in terms of bleeding reduction intraoperatively, transfusion rates, operation time, and cell saver volume.

The results of this meta-analysis are consistent with previous studies.^40,41^ Higher doses of TXA are generally associated with reduced bleeding and transfusion requirements during surgery.^40,41^ In terms of complications, the superiority of higher doses was not only statistically significant but also clinically relevant, as evidenced by lower bleeding and transfusion requirements associated with higher doses. A study conducted by Johnson confirmed these findings, clearly indicating that higher doses of TXA reduce bleeding and transfusion requirements.^29^ Moreover, the benefits of using higher doses of TXA extend beyond bleeding management. The pooled analysis of surgical time showed that TXA 4 had the highest P-scores in operation time and reduced cell saver transfusions compared with other TXA or TXA 0 groups. Optimization of the antifibrinolytic strategy may directly improve the efficacy of surgical procedures and the utilization of transfusion resources.

The TXA 3 group yielded the highest P-scores for postoperative and total blood loss, along with the lowest intraoperative and postoperative allogenic transfusion rates among all treatment groups. This suggests that higher TXA doses may lead to better management and potentially reduce transfusion-related complications among adolescent patients, including alloimmunization and other transfusion reactions.

Although the TXA 2 group had the shortest hospitalization length among all doses, there were no significant differences in complications. Moreover, other factors unrelated to TXA dosing could influence this outcome, so these results should be interpreted with caution.

The pharmacodynamics of higher doses of TXA generally support the dose-dependent effects observed. TXA acts as a competitive inhibitor of plasminogen activator and stabilizes fibrin clots. Higher doses of TXA may result in elevated plasma concentrations, which could be more beneficial in prolonged, extensive spinal surgeries, including the AIS posterior fusion procedure in teenagers.

However, while promising, this NMA has several limitations that must be acknowledged. Firstly, because there is no precise method to determine intraoperative blood loss. Blood loss estimation typically relies on a similar process that includes suction volume, surgical sponge weight, deduction of irrigation fluids, and serial hemoglobin values, which are conducted by the anesthesia and surgical teams. Secondly, we noted significant heterogeneity across some clinical outcomes, including blood loss or blood loss per vertebral level. Variations in study protocols, patient characteristics, perioperative care, and transfusion protocols may contribute to the inconsistency among different studies. Although a random-effects model was applied in this systematic analysis, demographic and protocol differences influenced the results. In addition, the bleeding outcome could be affected by oral contraceptives among female patients.

Moreover, while this study encompassed over 16 studies, only six randomized trials were included in this review. This study, therefore, primarily relied on comparisons from nonrandomized and retrospective studies. Furthermore, in the quality assessment (ROB2 and ROBINS-I), we observed moderate and severe concerns across the studies. More specifically, due to the lack of randomization, some studies exhibited bias in confounding or selecting participants. In addition, selective measurement of outcomes was present in both randomized and nonrandomized studies. The biases in the studies could impact the outcomes of this network analysis. Finally, the adverse events related to the dosing of TXA were not uniformly reported in the studies. Transfusion-related complications, such as thromboembolic events, were not analyzed because the studies did not report them. We assume this is likely due to the absence of such events in the included studies; however, since this cannot be definitively confirmed, we opted not to include this data. Although TXA appears to be safe, future studies must comprehensively assess the incidence of complications.

Notably, direct comparisons between some doses are still lacking, and future studies could consider comparing the currently missing doses. In addition, the cost-effectiveness of hospitalization could be directly compared and assessed to determine whether different TXA doses significantly impact hospitalization expenses. Moreover, long-term follow-up studies could provide more reliable insights for the analysis. Finally, the diversity in the number of studies in each arm was noted. For example, there were six studies in TXA 1 group, but only two studies in TXA 2 group. This aspect could affect the strength of the meta-analysis.

## CONCLUSIONS

The tranexamic acid dose of 100 mg/kg bolus plus 10 mg/kg/hour infusion (TXA 4) was the most effective for controlling bleeding in teenagers undergoing AIS surgery. TXA provides various perioperative benefits (transfusion rates, operation time, and cell saver volume) in the context of spinal fusion approaches. While these results are promising, additional randomized trials are needed to directly compare different TXA doses.

Key Points

- TXA is an antifibrinolytic agent widely used in spine surgery, including AIS instrumentation. However, the optimal TXA dose remains unclear.
- We evaluated five TXA doses: TXA 0 (control); TXA 1 (10 mg/kg bolus + 1 mg/kg/hour infusion); TXA 2 (15–20 mg/kg bolus + 1–10 mg/kg/hour infusion); TXA 3 (30–50 mg/kg bolus of TXA + 5–10 mg/kg/hour infusion); and TXA 4 (100 mg/kg bolus of TXA + 10 mg/kg/hour infusion).
- TXA 4 outperformed other doses in reducing bleeding, operation time, and cell saver volume. TXA 3 also showed significant benefits but was less effective than TXA 4. TXA 2 was associated with the shortest hospital stay.

## Supplementary Material

SUPPLEMENTARY MATERIAL
