# Supplementary material for: Optimal Tranexamic Acid Dosing for Adolescent Idiopathic Scoliosis Surgery: A Frequentist Network Meta-Analysis
Source: Spine (Phila Pa 1976). 2025 Aug 4;50(21):E438–48. doi: 10.1097/BRS.0000000000005465 (PMC12502950; doi:10.1097/BRS.0000000000005465)
Supplement: SUPPLEMENTARY MATERIAL [file brs-50-e438-s001.docx]

SDC Table 1: League table for intraoperative blood loss (PSF-only sensitivity analysis). Results are presented as mean differences with 95% CI

| TXA 0 |  |  |  |  |
| --- | --- | --- | --- | --- |
| 72.79 [ -43.05; 188.63]; p = 0.2181 | TXA 1 |  |  |  |
| 286.21 [ 87.62; 484.81]; p = 0.0047 | 213.43 [ -16.48; 443.34]; p = 0.0688 | TXA 2 |  |  |
| 152.52 [ 14.69; 290.34]; p = 0.0301 | 79.73 [ -61.85; 221.31]; p = 0.2697 | -133.70 [ -375.43; 108.04]; p = 0.2784 | TXA 3 |  |
| 934.58 [ 471.19; 1397.98]; p < 0.0001 | 861.80 [ 384.14; 1339.45]; p = 0.0004 | 648.37 [ 144.22; 1152.53]; p = 0.0117 | 782.07 [ 298.61; 1265.53]; p = 0.0015 | TXA 4 |
