# Supplementary material for: Optimal Tranexamic Acid Dosing for Adolescent Idiopathic Scoliosis Surgery: A Frequentist Network Meta-Analysis
Source: Spine (Phila Pa 1976). 2025 Aug 4;50(21):E438–48. doi: 10.1097/BRS.0000000000005465 (PMC12502950; doi:10.1097/BRS.0000000000005465)
Supplement: SUPPLEMENTARY MATERIAL [file brs-50-e438-s002.docx]

SDC Table 2: League table for total blood loss. Results are presented as mean differences with 95% CI

| TXA 0 |  |  |
| --- | --- | --- |
| 782.71 [ 498.99; 1066.43]; p < 0.0001 | TXA 1 |  |
| 805.85 [ 552.53; 1059.18]; p < 0.0001 | 23.14 [ -164.16; 210.44]; p = 0.8087 | TXA 3 |
