# Supplementary material for: Optimal Tranexamic Acid Dosing for Adolescent Idiopathic Scoliosis Surgery: A Frequentist Network Meta-Analysis
Source: Spine (Phila Pa 1976). 2025 Aug 4;50(21):E438–48. doi: 10.1097/BRS.0000000000005465 (PMC12502950; doi:10.1097/BRS.0000000000005465)
Supplement: SUPPLEMENTARY MATERIAL [file brs-50-e438-s003.docx]

SDC Table 3: League table for intraoperative blood loss per level. Results are presented as mean differences with 95% CI

| TXA 0 |  |  |  |
| --- | --- | --- | --- |
| 18.17 [ -7.80; 44.14]; p = 0.1703 | TXA 1 |  |  |
| 21.25 [ -2.76; 45.26]; p = 0.0828 | 3.08 [ -20.33; 26.49]; p = 0.7963 | TXA 3 |  |
| 192.82 [ 107.70; 277.94]; p < 0.0001 | 174.65 [ 85.66; 263.64]; p = 0.0001 | 171.57 [ 83.13; 260.01]; p = 0.0001 | TXA 4 |
