# Supplementary material for: Optimal Tranexamic Acid Dosing for Adolescent Idiopathic Scoliosis Surgery: A Frequentist Network Meta-Analysis
Source: Spine (Phila Pa 1976). 2025 Aug 4;50(21):E438–48. doi: 10.1097/BRS.0000000000005465 (PMC12502950; doi:10.1097/BRS.0000000000005465)
Supplement: SUPPLEMENTARY MATERIAL [file brs-50-e438-s004.docx]

SDC Table 4: League table for length of hospitalization. Results are presented as mean differences with 95% CI

| TXA 0 |  |  |  |
| --- | --- | --- | --- |
| -0.19 [-0.57; 0.19]; p = 0.3273 | TXA 1 |  |  |
| 0.77 [-0.08; 1.62]; p = 0.0744 | 0.96 [ 0.03; 1.88]; p = 0.0425 | TXA 2 |  |
| 0.07 [-0.28; 0.42]; p = 0.6959 | 0.26 [-0.12; 0.64]; p = 0.1862 | -0.70 [-1.62; 0.21]; p = 0.1335 | TXA 3 |
