# Supplementary material for: Optimal Tranexamic Acid Dosing for Adolescent Idiopathic Scoliosis Surgery: A Frequentist Network Meta-Analysis
Source: Spine (Phila Pa 1976). 2025 Aug 4;50(21):E438–48. doi: 10.1097/BRS.0000000000005465 (PMC12502950; doi:10.1097/BRS.0000000000005465)
Supplement: SUPPLEMENTARY MATERIAL [file brs-50-e438-s005.docx]

SDC Table 5: League table for volume (ml) of Intraoperative Cell Saver Transfusion. Results are presented as mean differences with 95% CI

| TXA 0 |  |  |  |
| --- | --- | --- | --- |
| 289.30 [ 276.41; 302.19]; p < 0.0001 | TXA 2 |  |  |
| 47.81 [ 17.53; 78.10]; p = 0.0020 | -241.49 [ -274.40; -208.57]; p < 0.0001 | TXA 3 |  |
| 1108.90 [ 685.84; 1531.96]; p < 0.0001 | 819.60 [ 396.35; 1242.85]; p = 0.0001 | 1061.09 [ 636.95; 1485.23]; p < 0.0001 | TXA 4 |
