# Supplementary material for: Optimal Tranexamic Acid Dosing for Adolescent Idiopathic Scoliosis Surgery: A Frequentist Network Meta-Analysis
Source: Spine (Phila Pa 1976). 2025 Aug 4;50(21):E438–48. doi: 10.1097/BRS.0000000000005465 (PMC12502950; doi:10.1097/BRS.0000000000005465)
Supplement: SUPPLEMENTARY MATERIAL [file brs-50-e438-s006.docx]

SDC Table 6: League table for intraoperative allogenic transfusion rate. Results are presented as odds ratio with 95% CI

| TXA 0 |  |  |  |
| --- | --- | --- | --- |
| 0.60 [0.29; 1.27]; p = 0.1810 | TXA 1 |  |  |
| 1.81 [0.25; 13.23]; p = 0.5612 | 3.00 [0.47; 19.04]; p = 0.2439 | TXA 2 |  |
| 1.85 [1.03; 3.35]; p = 0.0405 | 3.08 [1.47; 6.45]; p = 0.0028 | 1.03 [0.14; 7.51]; p = 0.9786 | TXA 3 |
