# Supplementary material for: Optimal Tranexamic Acid Dosing for Adolescent Idiopathic Scoliosis Surgery: A Frequentist Network Meta-Analysis
Source: Spine (Phila Pa 1976). 2025 Aug 4;50(21):E438–48. doi: 10.1097/BRS.0000000000005465 (PMC12502950; doi:10.1097/BRS.0000000000005465)
Supplement: SUPPLEMENTARY MATERIAL [file brs-50-e438-s007.docx]

SDC Table 7: League table for postoperative allogenic transfusion rate. Results are presented as odds ratio with 95% CI

| TXA 0 |  |  |
| --- | --- | --- |
| 0.74 [0.17; 3.20]; p = 0.6915 | TXA 1 |  |
| 3.46 [1.72; 6.98]; p = 0.0005 | 4.66 [0.92; 23.50]; p = 0.0626 | TXA 3 |
