# Supplementary material for: Optimal Tranexamic Acid Dosing for Adolescent Idiopathic Scoliosis Surgery: A Frequentist Network Meta-Analysis
Source: Spine (Phila Pa 1976). 2025 Aug 4;50(21):E438–48. doi: 10.1097/BRS.0000000000005465 (PMC12502950; doi:10.1097/BRS.0000000000005465)
Supplement: SUPPLEMENTARY MATERIAL [file brs-50-e438-s009.docx]

SDC Table 9: League table for complications (PSF-only sensitivity analysis). Results are presented as odds ratio with 95% CI

| TXA 0 |  |  |  |  |
| --- | --- | --- | --- | --- |
| 1.64 [0.33; 8.29]; p = 0.5471 | TXA 1 |  |  |  |
| 3.02 [0.25; 36.69]; p = 0.3858 | 1.84 [0.13; 25.52]; p = 0.6507 | TXA 2 |  |  |
| 0.79 [0.10; 6.20]; p = 0.8253 | 0.48 [0.10; 2.27]; p = 0.3564 | 0.26 [0.01; 5.12]; p = 0.3778 | TXA 3 |  |
| 1.30 [0.08; 20.99]; p = 0.8552 | 0.79 [0.03; 19.74]; p = 0.8849 | 0.43 [0.01; 18.08]; p = 0.6576 | 1.63 [0.05; 52.05]; p = 0.7811 | TXA 4 |
