# Supplementary material for: Optimal Tranexamic Acid Dosing for Adolescent Idiopathic Scoliosis Surgery: A Frequentist Network Meta-Analysis
Source: Spine (Phila Pa 1976). 2025 Aug 4;50(21):E438–48. doi: 10.1097/BRS.0000000000005465 (PMC12502950; doi:10.1097/BRS.0000000000005465)
Supplement: SUPPLEMENTARY MATERIAL [file brs-50-e438-s010.docx]

SDC Table 10: Summary of P-scores across all outcomes in 6-arm NMA

| Outcome | Heterogeneity | TXA 0 | TXA 1 | TXA 2 | TXA 3A | TXA 3B | TXA 4 |
| --- | --- | --- | --- | --- | --- | --- | --- |
| Intraoperative blood loss | 67.80% | 0.1269 | 0.2963 | 0.7461 | 0.2239 | 0.6124 | **0.9943** |
| Total blood loss | 0.00% | 0.0047 | 0.5648 | N/A | 0.5184 | **0.912** | N/A |
| Postoperative blood loss | 0.00% | 0.0006 | 0.6376 | 0.254 | **0.9111** | 0.6967 | N/A |
| Blood loss per level | 58.20% | 0.0881 | 0.4355 | N/A | 0.237 | 0.7395 | **1** |
| Operation time | 55.40% | 0.2468 | 0.6366 | 0.5963 | 0.1239 | 0.4035 | **0.9929** |
| Complications | 0.00% | 0.3814 | 0.5972 | **0.7198** | 0.5395 | 0.2825 | 0.4797 |
